# Dyslexia risk variant rs600753 is linked with dyslexia-specific differential allelic expression of *DYX1C1*

**DOI:** 10.1590/1678-4685-GMB-2017-0165

**Published:** 2018-02-19

**Authors:** Bent Müller, Johannes Boltze, Ivonne Czepezauer, Volker Hesse, LEGASCREEN Consortium, Arndt Wilcke, Holger Kirsten

**Affiliations:** 1 Fraunhofer Institute for Cell Therapy and Immunology, Leipzig, Germany; 2 Fraunhofer Research Institution for Marine Biotechnology, Department of Medical Cell Technology, Lübeck, Germany; 3 Institute for Medical and Marine Biotechnology, University of Lübeck, Lübeck, Germany; 4 German Center for Growth, Development and Health Encouragement in Childhood and Adolescence, Berlin, Germany; 5 Charité-University Medicine Berlin, Institute for Experimental Paediatric Endocrinolgy, Berlin; 6 Institute for Medical Informatics, Statistics and Epidemiology, University of Leipzig, Leipzig, Germany; 7 LIFE - Leipzig Research Center for Civilization Diseases, University of Leipzig, Leipzig, Germany

**Keywords:** dyslexia, SNP, eQTL, differential allelic expression

## Abstract

An increasing number of genetic variants involved in dyslexia development were discovered during the last years, yet little is known about the molecular functional mechanisms of these SNPs. In this study we investigated whether dyslexia candidate SNPs have a direct, disease-specific effect on local expression levels of the assumed target gene by using a differential allelic expression assay. In total, 12 SNPs previously associated with dyslexia and related phenotypes were suitable for analysis. Transcripts corresponding to four SNPs were sufficiently expressed in 28 cell lines originating from controls and a family affected by dyslexia. We observed a significant effect of rs600753 on expression levels of *DYX1C1* in forward and reverse sequencing approaches. The expression level of the rs600753 risk allele was increased in the respective seven cell lines from members of the dyslexia family which might be due to a disturbed transcription factor binding sites. When considering our results in the context of neuroanatomical dyslexia-specific findings, we speculate that this mechanism may be part of the pathomechanisms underlying the dyslexia-specific brain phenotype. Our results suggest that allele-specific *DYX1C1* expression levels depend on genetic variants of rs600753 and contribute to dyslexia. However, these results are preliminary and need replication.

## Introduction

Dyslexia is a highly heritable disorder. The genetic component contributes by up to 60% to this disorder (Schulte-Körne, 2010) and several genes are suggested to affect the development of dyslexia (see Tables S1 and S2). Variants of well-validated genes such as *DCDC2* (Doublecortin Domain Containing 2), *KIAA0319*, *ROBO1* (Roundabout Guidance Receptor 1) and *DYX1C1* (Dyslexia Susceptibility 1 Candidate 1) are believed to be involved in disturbed neuronal migration and axonal guidance (Carrion-Castillo *et al.*, 2013) as well as differences of brain phenotypes such as alterations in white matter structure (Darki *et al.*, 2012). In contrast to relatively well-established knowledge on the gene level, information regarding the molecular mechanisms of dyslexia candidate single nucleotide polymorphisms (SNPs) is still fragmentary.

The molecular mechanism exerted by a certain SNP can be of different nature. On the one hand, SNPs might affect the structure of a gene-derived protein. For dyslexia, however, only very few nonsynonymous SNPs affecting protein structure and function are known (see Table S2 for an overview). On the other hand, SNPs might influence the protein quantitatively, e.g., by altering gene expression levels, a phenomenon also referred to as expression quantitative loci (eQTL). eQTLs are commonly differentiated in *cis-*, as well as in *trans-*acting eQTLs. *Trans* eQTLs are located distant to the gene which expression is affected and *cis* eQTLs are located close to the affected gene.

Only few studies specifically analyzed the impact of dyslexia candidate SNPs on gene expression levels. Two groups (Tapia-Páez *et al.*, 2008; Tammimies *et al.*, 2012) reported a putative effect of rs3743205 on expression level of *DYX1C1*. Tammimies *et al.* (2012) observed that a CpG site results from the G-allele of this SNP. This might lead to methylation of a transcription factor binding site, and, consequently, in disturbed binding of transcription factors. Paracchini *et al.* (2006) observed reduced expression levels of *KIAA0319* in carriers of the risk haplotype rs4504469-rs2038137-rs2143340. In a second study, Dennis *et al.* (2009) tested seven SNPs of the *KIAA0319* promotor region and observed reduced *KIAA01319* expression levels for the minor allele rs9461045-T. However, these experiments were all carried out in cells derived from donors without dyslexia background. Such approaches might miss effects resulting from a disease-specific genetic background. Previous results from dyslexia, but also from other complex diseases, motivate to analyze such effects. For example, Hannula-Jouppi *et al.* (2005) observed disease-specific gene-expression levels in dyslexia and Furney *et al.* (2011) identified an Alzheimer-specific effect for a SNP within *ZNF292* on entorhinal cortical volume. A disease-specific molecular mechanisms can be understood as an effect which only emerges in affected individuals. This might be due to different regulatory networks present in the affected individuals. Consequently, certain molecular factor might be active in the affected individuals, only, e.g., certain transcription factors. If a certain SNP would alter the genomic binding site of such a disease specific factor, the effect of this SNP would be also disease-specific, i.e., observed only in the affected individuals (de la Fuente, 2010).

Another problem that may affect detection of an effect of a genetic variant on gene expression might result from general heterogeneity across samples from a variety of biological and technical sources, what can decrease study power. However, these limitations can be addressed by the direct measurement of *cis*-regulated allelic expression differences by differential allelic expression (DAE) (Serre *et al.*, 2008). In this approach, expression differences resulting from the two different alleles of a SNP are analyzed within heterozygous individuals. Consequently, this method is rather robust to biological or technical batch effects among individuals.

In our study we conducted a stepwise approach to identify dyslexia-specific effects of SNPs on gene expression. We started with the identification of suitable dyslexia candidate SNPs having a potential effect on local gene expression levels by assigning functional properties (McLaren *et al.*, 2010) (Tables S1 and S2). Subsequently, all these SNPs were genotyped in 10 cell lines derived from multiple members of a family in which dyslexia frequently occurred, thus providing a disease-specific background, and in 18 control cell lines. Disease-specific DAE was assessed in two replicates applying forward and reverse Sanger sequencing of reverse transcribed cDNA. Results were compared with publically available (dyslexia-unspecific) eQTL-data.

## Materials and Methods

### SNP selection, cell lines and characterization steps

For the identification of dyslexia candidate SNPs, we conducted a systematic screening using ‘PubMED’ and ‘Google Scholar’ for genetic candidate-studies related to dyslexia. The identified SNPs had to map to an exonic, 5’-UTR or 3’-UTR location to have the potential to affect local expression levels of the target genes. For each of these SNPs, a minimum of four heterozygous cell lines per group was required to maintain validity of our analyses (Serre *et al.*, 2008), and the general (non-allele specific) expression of the SNP-corresponding transcripts was tested with cDNA-specific primers in the sample cells of interest.

In total, 28 Epstein-Barr virus (EBV) immortalized cell lines derived from B-cells were available. Ten cell lines were derived from a three generational German family, in which dyslexia segregation suggests a full-penetrance, autosomal dominant inheritance. A genome-wide linkage analysis revealed a haplotype of chromosome 12 co-segregating with language impairment (Addis *et al.*, 2010). For a detailed description of the family see Addis *et al.* (2010). 18 cell lines that served as controls were derived from several families with more details available elsewhere (Burkhardt *et al.*, 2012).

Extraction of genomic DNA (DNeasy Blood & Tissue Kit, Qiagen, Hilden, Germany) and subsequent genotyping was performed by the matrix-assisted laser desorption/ionization time-of-flight spectrometry system iPLEX (Agena, Hamburg, Germany). SNPs had to fulfill Hardy-Weinberg-Equilibrium criteria (HWE; *p* > 0.05 after Bonferroni correction), and to exhibit a SNP-wise call rate > 97%, as well as a minor allele frequency (MAF) > 0.05.

**Figure 1:**
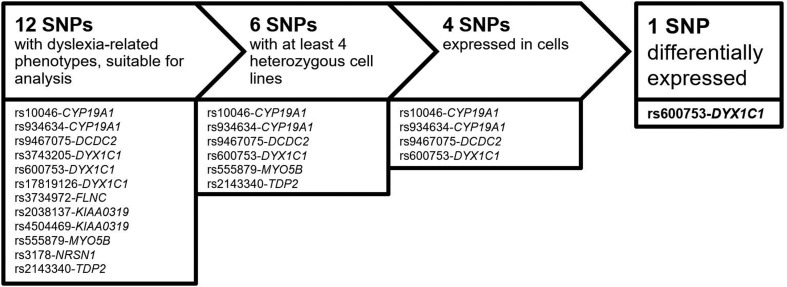
Workflow of SNP characterization. Candidate SNPs used in this study had to be heterozygous in at least three immortalize B-cell lines originating from the dyslexia family and three cell lines originating from controls and expressed in the immortalized B-cells.

SNPs had to be heterozygous in at least four individual cell lines to be eligible for analysis. Six SNPs fulfilled this criterion and were considered for further analyses. Appropriate cDNA-specific primers were designed and tested for blood-specific expression. Gel electrophoresis demonstrated sufficient expression of four SNPs in B-cells and were therefore analyzed for DAE. Figure 1 illustrates the workflow.

**Figure 2:**
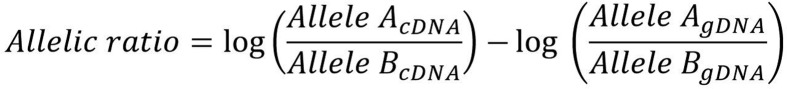
Formula for calculating the log-transformed allelic ratio. The allelic ratio is the difference between the natural logarithm-transformed ratios of the allele heights of the cDNA and the gDNA.

Heterozygous samples were quantified on cDNA and gDNA level. cDNA was reverse-transcribed from RNA using Direct-zol RNA MiniPrep (Zymo Research, Irvine, CA, USA) and Oligo(dT)_15_ primer (Promega, Madison, Wisconsin, USA). Exonic, cDNA-specific PCR primers, and gDNA-specific, intronic PCR primers were designed as flanking the four SNPs. PCR was carried out with 45 rounds and 58 °C annealing temperature. For further details, see Wilcke *et al.* (2009) and Müller *et al.* (2016). To quantify DAE, genomic and coding PCR products harboring the SNP of interest were purified (Qiaquick PCR Purification Kit, Qiagen, Hilden, Germany) and Sanger-sequenced by Seqlab (Göttingen, Germany). Relative peak heights of the SNP of interest were quantified using R software version 3.2.4 (R Core Team, 2016) applying the add-on package sangerseqR version 1.4.0 (Hill *et al.*, 2014). Allelic ratios were calculated and log-transformed (see Figure 2). For each SNP, the log-transformed allelic ratio was corrected in an assay-specific manner (forward or reverse) by subtracting the respective average transformed gDNA ratio. Primer sequences can be found in Table S3.

### Statistical analyses

To identify a genetic effect on gene expression, we used the Kruskal-Wallis test to analyze significant differences between the allelic ratios of three groups: (i) affected dyslexia family, (ii) controls, and (iii) gDNA. We used the pairwise Wilcoxon rank sum test as post-hoc test, applying the closed test procedure to account for multiple testing. Results from forward and reverse sequencing were analyzed separately as well as combined by averaging allelic log-ratios.

### *In silico* characterization and comparison with reported eQTLs

SNPs were characterized *in silico* according to Ensembl annotations and prediction data (McLaren *et al.*, 2010). Furthermore, SNPs were annotated with known and predicted regulatory elements including binding sites of transcription factors and promoter regions using RegulomeDB (Boyle *et al.*, 2012). Publication-based (dyslexia-unspecific) eQTLs comparison was performed by screening 24 published eQTL datasets (Dixon *et al.*, 2007; Myers *et al.*, 2007; Veyrieras *et al.*, 2008; Heinzen *et al.*, 2008; Ding *et al.*, 2010; Liu *et al.*, 2010; Murphy *et al.*, 2010; Zeller *et al.*, 2010; Gibbs *et al.*, 2010; Barreiro *et al.*, 2011; Borel *et al.*, 2011; Fehrmann *et al.*, 2011; Grundberg *et al.*, 2011; Kompass and Witte, 2011; Qiu *et al.*, 2011; Innocenti *et al.*, 2011; Xia *et al.*, 2012; Zou *et al.*, 2012; Kim *et al.*, 2012; Kabakchiev and Silverberg, 2013; Westra *et al.*, 2013; Ramasamy *et al.*, 2014; Kirsten *et al.*, 2015; GTEx Consortium, 2015). These publications cover a broad range of 63 different tissues, including brain and neuronal tissues, as well as *cis* and *trans* eQTL data.

The LD structure of the *DYX1C1* locus was analyzed via local association plots using LocusZoom software (Pruim *et al.*, 2011).

## Results

### Functional variant annotation and identification of eligible SNPs

We identified 12 suitable SNPs with reported associations with dyslexia related phenotypes that have the potential to affect local expression levels: rs934634-*CYP19A1*, rs10046*-CYP19A1* and rs555879-*MYO5B* are located in the 3’-UTR, rs3743205- *DYX1C1*, rs2038137-*KIAA0319* and rs3178*-NRSN1* are located in the 5’-UTR. Rs600753-*DYX1C1*, rs17819126-*DYX1C1*, rs9467075-*DCDC2*, rs3734972*-FLNC* and rs4504469-*KIAA0319* are exonic SNPs, and rs2143340-*TDP2* is located in a non-coding exon. Genotyping of these SNPs was performed in all 28 cell lines in order to identify heterozygous cell lines.

In a second step, SNPs were only considered for analyses if a minimum of three heterozygous cell lines from the dyslexia family as well as control cell lines were available. Six of the preselected SNPs fulfilled this criterion (rs10046*-CYP19A1* (6 dyslexia and 11 controls), rs934634-*CYP19A1* (4 dyslexia and 9 controls), rs9467075-*DCDC2* (5 dyslexia and 8 controls), rs600753-*DYX1C1* (7 dyslexia and 10 controls), rs555879-*MYO5B* (7 dyslexia and 14 controls), rs2143340-*TDP2* (4 dyslexia and 7 controls)).

In a third step, sufficient expression of the transcripts corresponding to the SNPs in EBV cells was tested by cDNA-specific PCR. Four SNPs fulfilled these three criteria (rs10046*-CYP19A1*, rs600753-*DYX1C1*, rs934634-*CYP19A1*, rs9467075-*DCDC2*) and, thus, were tested for dyslexia-specific effects on gene expression (Figure 1).

Finally, sequences must have passed quality control. Hence, for rs600753, data from up to six dyslexics and seven controls, were included in differential allelic expression analysis. For detailed numbers see Table S4.

### Differential allelic expression

**Figure 3:**
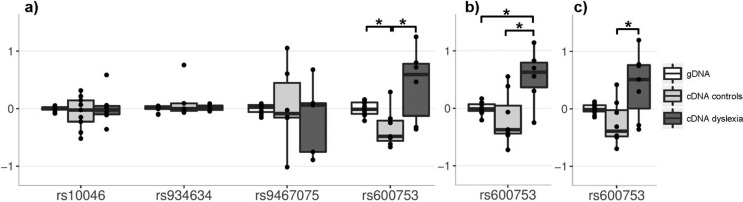
Differential allelic expression (DAE) of four dyslexia-related SNPs. Shown are allelic log-ratios measured in heterozygous samples adjusted for the gDNA allelic ratio. a) Forward sequencing analysis of all four expressed SNPs stratified for cDNA allelic ratios for controls and the dyslexia affected family and the logarithm of the gDNA allelic ratio. b) Reverse sequencing based replication of rs600753. c) Analysis of both sequencing approaches together. *p < 0.05 (Wilcoxon rank sum test). Global testing for genetic effects on gene expression of rs600753-*DYX1C1* applying Kruskal-Wallis test was always *p* < 0.05. For details see Table 1.

**Figure 4:**
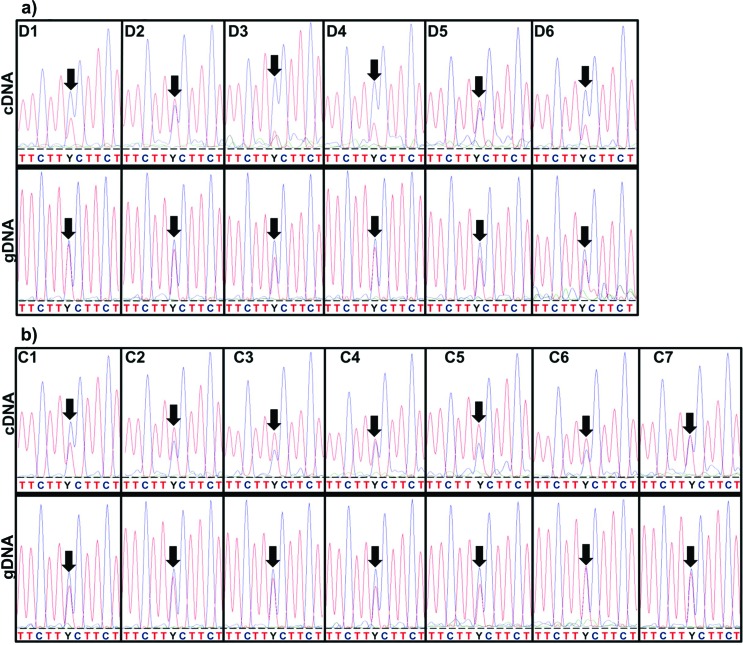
Sequencing results of dyslexia family and controls. The rs600753 cDNA-sequences and the respective gDNA-sequences for six dyslexia family members (a) and seven controls (b). Arrows indicate position of rs600753.

No genetic effects on gene expression were observed for variants rs10046*-CYP19A1*, rs934634-*CYP19A1*, rs9467075-*DCDC2.* In contrast, we observed a significant effect of rs600753 on *DYX1C1* expression levels (Table 1 and Figure 3; raw-data is shown in Figure 4). In particular, an effect of rs600753 on the forward sequencing based measurement was observed (*p*=0.016). The post-hoc test revealed significant differences between the cDNA levels of the dyslexia family and the controls. The significant difference could be confirmed in data from reverse sequencing (*p*=0.013), as well as within the combined analyses of both approaches (*p*=0.021). This showed that the reported dyslexia risk-allele (rs600753-C) was expressed higher than the protective allele (T) in the dyslexia family. The control cell lines revealed the opposite effect, as the T-allele was higher expressed compared to the C-allele (Table 1).

**Table 1:** Association statistics of the four dyslexia-related SNPs and DAE.

SNP	Sequencing direction (allelic ratio)	Mean allelic ratios (SD)			Global p-value (Kruskal-Wallis test)	Post-hoc p-values		
		gDNA	cDNA controls	cDNA dyslexia		cDNA control vs. cDNA dyslexia	cDNA dyslexia vs. gDNA	cDNA controls vs. gDNA
rs10046*-CYP19A1*	F (C/T)	0 (0.04)	-0.07 (0.28)	0.02 (0.31)	0.937			
rs600753-*DYX1C1*	F (C/T)	0 (0.11)	-0.34 (0.33)	0.42 (0.64)	0.016*	0.035*	0.2672	0.0085*
rs934634-*CYP19A1*	F (G/A)	0 (0.06)	0.15 (0.35)	0.02 (0.06)	0.917			
rs9467075-*DCDC2*	F (A/G)	0 (0.08)	0.05 (0.71)	-0.16 (0.65)	0.906			
rs600753- *DYX1C1*	R (G/A)	0 (0.10)	-0.19 (0.44)	0.55 (0.48)	0.013*	0.013*	0.024*	0.115
rs600753- *DYX1C1*	F&R	0 (0.08)	-0.25 (0.37)	0.41 (0.57)	0.027*	0.021*	0.123	0.065

‘F’ denotes forward sequencing, ‘R’ denotes reverse sequencing replication and ‘F+R’ denotes analysis of both approaches together. Shown are the logarithm of the cDNA allelic ratios for controls and the dyslexia affected family members, the logarithm of the gDNA allelic ratio and the order the ratios were formed. P-values are given for the Kruskal-Wallis test comparing transformed allelic ratios of three groups (allelic ratios of cDNA from cell lines originating from the dyslexia family members, allelic ratios from cDNA from cell lines originating from controls and allelic ratios from gDNA). P-values ≤ 0.05 are indicated by an asterisk.

Since a single previous study reported sex-specific association of rs600753 with dyslexia (Dahdouh *et al.*, 2009), we stratified our DAE analysis of rs600753 for sex. However, we did not observe any sex-specific effect. The risk and non-risk individuals revealed a similar genetic effect on gene expression for both sexes.

### Functional annotation

Rs600753 was annotated with regulatory elements using RegulomeDB which includes the identification of transcription factor binding sites and their disturbance by position weight matrix (PWM). PWM indicates the disturbance of binding sites the transcription factor *Srf*, *Nanog*, *Mtf1* by rs600753 (Matys *et al.*, 2006; Badis *et al.*, 2009; Boyle *et al.*, 2012). Furthermore, the publication-based annotation with eQTL-effects revealed an *cis* effect of rs600753 on the expression levels of *CCPG1* and *PIGB* in blood derived cells (Xia *et al.*, 2012; Westra *et al.*, 2013; Kirsten *et al.*, 2015), and *DYX1C1* in fibroblasts (GTEx Consortium, 2015).

## Discussion

This study analyzed dyslexia candidate SNPs with regard to their disease-specific effect on the expression levels of their respective gene. This was performed by applying a DAE analysis of cells from a dyslexia family and controls. SNP rs600753 indicated an effect on the expression level of *DYX1C1* with the reported risk allele rs600753-C (Dahdouh *et al.*, 2009; Matsson *et al.*, 2015) being stronger expressed in cell lines derived from a dyslexia family as compared to cell lines from controls.

Association of rs600753 with dyslexia related phenotypes was first reported by Dahdouh *et al.* (2009) who identified an association of a haplotype spanning rs3743205, rs3743204 and rs600753 in females. Variant rs600753 efficiently tags this haplotype. Corroborating, a nominally significant single-marker association of SNP rs600753 with spelling was identified in German dyslexia families (Matsson *et al.*, 2015). Our study further supports a role of rs600753 in dyslexia as we found disease-specific effects of rs600753 on expression levels of *DYX1C1*.

A distinction must be drawn between disease-specific effects, such as those investigated here, and general, non-disease-specific effects. The disease-specific effect of rs600753 identified in this study can be explained by the complex genetic background underlying the disease. Affected individuals might exhibit changes in regulatory networks. This may lead, e.g., to the activation of transcription factors that are not active in unaffected controls. If a SNP causes a differential allelic expression of a binding site of a such factor, the effect of the SNP can be disease-specific. To the best of our knowledge, there is only one report of a SNP affecting gene expression levels in a dyslexia-specific manner. Hannula-Jouppi *et al.* (2005) observed allele-specific expression of a SNP in the 3’-UTR of *ROBO1* (6483T > A) in a dyslexic Finnish family. The expression of the A-allele was absent or attenuated in four individuals. However, the same group was unable to directly replicate this finding in a more recent study (Massinen *et al.*, 2016), but the conclusion remained that adequate *ROBO1* expression is a prerequisite for a normal crossing of the auditory pathway (Lamminmäki *et al.*, 2012; Massinen *et al.*, 2016). We aimed to control for such variations by analyzing both strands.

Other studies investigated an effect of dyslexia candidate SNPs on gene expression levels in non-dyslexic samples, only. For instance, two studies reported effects of rs3743205 on the expression levels of *DYX1C1* (Tapia-Páez *et al.*, 2008; Tammimies *et al.*, 2012). Similar results were reported for *KIAA0319.* Reduced expression levels were observed for the haplotype rs4504469-rs2038137-rs2143340 (Paracchini *et al.*, 2006) and for rs9461045 (Dennis *et al.*, 2009). We tested the expression of *KIAA0319* with rs2038137, yet this gene was not expressed in the available cell lines. Furthermore, risk allele frequency of rs3743205-*DYX1C1* was not sufficient in our sample. Consequently, from our study we cannot draw conclusions concerning genetic effects on gene expression of these SNPs.

In cell lines from the dyslexia family we observed significantly increased expression associated with the reported risk variant rs600753-C. Thereby we observed individual differences in the effect size (Figures 2), which is potentially due to a number of reasons. These include environmental factors and the presence of additional genetic factors modulating *DYX1C1* expression levels. It certainly would be of interest to see whether a generally increased *DYX1C1* expression level is present in the investigated dyslexia family including the non-carriers of the rs600753 risk variants. However, this comparison is not available due to the low number of available homozygous individuals.

The impact of altered gene expression levels on neuronal function was repeatedly observed for the best replicated dyslexia candidate genes (*DCDC2, DYX1C1* and *KIAA0319*). Knockdown experiments for these genes in rats revealed disrupted neuronal migration to the neocortex (Adler *et al.*, 2013). Particularly, neurons from *Dyx1c1* knockdown rats exhibited bimodal ectopic locations by remaining at the white matter border or migrating beyond their expected position (Currier *et al.*, 2011). Similar ectopic neuronal locations were also observed in brains of dyslexic individuals (Galaburda *et al.*, 1985). We speculate that rs600753-*DYX1C1* is part of the pathomechanism underlying the characteristic dyslexia phenotype described by Galaburda *et al.* (1985): Expression levels of genes being relevant for neurogenesis need to be strictly controlled, and too low as well as too high expression can be deleterious (Francesconi and Lehner, 2014). Thus, allele-specific alterations of *DYX1C1* expression levels linked to rs600753 might have the potential to disturb downstream effects of *DYX1C1,* such as neuronal migration and neuronalplacement, and thereby affecting functionality of the resulting neural networks.

PWM-assays support this hypotheses as they indicated a disturbance of binding sites of three different transcription factors (*Srf*, *Nanog*, *Mtf1*) by rs600753 (Matys *et al.*, 2006; Badis *et al.*, 2009; Boyle *et al.*, 2012). Altered binding of these transcription factors might provide a molecular mechanism for the observed genetic regulation. *Srf* (OMIM 600589) is an ubiquitous nuclear protein known to be involved in cell growth, *Mtf1* (OMIM 600172) is involved in metal homeostasis and *Nanog* (OMIM 607937) is involved in embryonic stem cell proliferation and renewal. Hence, among these three putative affected transcription factors we consider *Nanog* as the most interesting candidate in the context of the molecular pathomechanism of dyslexia as its function provides a direct link to early developmental processes critical in dyslexia.

We analyzed published eQTL-data of unaffected populations to obtain further insights into the observed rs600753-*DYX1C1* effect. Rs600753 directly affects the expression levels of *CCPG1* and *PIGB* in blood derived cells (Xia *et al.*, 2012; Westra *et al.*, 2013; Kirsten *et al.*, 2015), and *DYX1C1* in fibroblasts (GTEx Consortium, 2015). The reported effect direction is in line with the direction we observed for the control cell lines (higher expressed T-allele). This strengthens the hypothesis that the effect of rs600753 is dyslexia-specific since we observed a significant opposite effect direction in cells from the dyslexia family (higher expressed C-allele).

However, rs600753 is not the strongest reported eQTL at this locus (Figure S1), as reported effects of rs12324434 are stronger (*p*=4.510^-16^). This variant is in moderate linkage disequilibrium (R^2^=0.67) with rs600753 (GTEx Consortium, 2015). Notably, two studies analyzed an association of rs12324434 with dyslexia but found no association (Bates *et al.*, 2010; Paracchini *et al.*, 2011). Therefore, in contrast to rs600753, a putative relevance of rs12324434 for dyslexia remains to be shown.

### Limitations

We investigated DAE in immortalized B-cells and not in neuronal cell lines. However, it is well known that most *cis* eQTL are ubiquitous, as typically more than 50% are replicable among tissues (Van Nas *et al.*, 2010; GTEx Consortium, 2015). All investigated affected individuals originated from a single, large dyslexia family, which limits the generalizability of our observation. Hence, our findings should be considered as preliminary and provocative and should be replicated in larger numbers of affected and unaffected individuals. Nevertheless, this family was very well characterized for any medical conditions, and dyslexia was the primary characteristic. Hence, we expect that the described genetic effects on gene expression is likely of dyslexia-specific nature. In line with this, when stratifying our data of rs600753 for affection with dyslexia, an even higher DAE was observed in cells originating from family members with reported dyslexia compared with family members without reported dyslexia (Figure S2). Moreover, all investigated cell lines originated from individuals of Caucasian ancestry. Although this eliminates an important source of false positives due to population stratification, this limits at the same time the transferability of our findings to other ethnicities.

## Conclusion

We identified allele-specific *DYX1C1* expression levels related to rs600753 in dyslexics, a variant previously reported to be associated with dyslexia. Our findings are among the first for dyslexia candidate SNPs suggesting an effect on gene expression in a dyslexia-specific manner. The results are in line with reported eQTL data and provide further insights into the molecular pathomechanisms of dyslexia.

## Supplementary material

The following online material is available for this article:

Table S1 Overview of considered SNPs.

Table S2 Overview of SNPs related to dyslexia but not analyzed.

Table S3 Primer sequences.

Table S4 Number of sequences surviving quality control.

Figure S1 Local association plot of rs600753.

Figure S2 The effect of rs600753 stratified for gDNA
