# Supplementary material for: Dyslexia risk variant rs600753 is linked with dyslexia-specific differential allelic expression of DYX1C1
Source: Genet Mol Biol. 2018 Feb 19;41(1):41–9. doi: 10.1590/1678-4685-GMB-2017-0165 (PMC5901500; doi:10.1590/1678-4685-GMB-2017-0165)
Supplement: Supplementary file 1 [file 1415-4757-GMB-41-01-2017-0165-s002.pdf]

Supplementary material to "Dyslexia risk variant rs600753 is linked with dyslexia-specific differential allelic expression of *DYX1C1*"

Table S2 - Overview of SNPs related to dyslexia but not analyzed.

SNP	Chr	Position	Gene	Type	Epidemiological evidence	SNP properties
**rs196402**	1	24838493	*RCAN3*	A/G	Luciano et al. 2013	intron
**rs1000585**	2	75823162	*AC005034.2*	A/G	Anthoni et al. 2007; Newbury et al. 2011	near gene
**rs917235**	2	75825819	*AC005034.2*	A/G	Anthoni et al. 2007	near gene
**rs714939**	2	75835107	*EVA1A*	A/G	Anthoni et al. 2007	-
**rs6732511**	2	75839733	*MRPL19*	C/T	Anthoni et al. 2007	-
**rs730148**	2	76357815	*SUCLA2P2*	C/G	Anthoni et al. 2007	-
**rs991684**	2	208099497	*AC007879.2*	A/G	Harlaar et al. 2005	intron
**rs12636438**	3	22063277	*ZNF385D*	A/G	Eicher et al. 2013	intron
**rs331142**	3	78920844	*ROBO1*	A/G/T	Tran et al. 2014	intron
**rs12495133**	3	78921520	*ROBO1*	A/C	Tran et al. 2014	intron
**rs6802848**	3	79411014	*ROBO1*	A/G	Bates et al. 2011	intron
**rs1387665**	3	79429811	*ROBO1*	A/G	Bates et al. 2011	intron
**rs4680960**	3	79449566	*ROBO1*	C/T	Bates et al. 2011	intron
**rs4130991**	3	79469022	*ROBO1*	A/G	Bates et al. 2011	intron
**rs4264688**	3	79546348	*ROBO1*	A/G	Bates et al. 2011	intron
**rs7622444**	3	79557927	*ROBO1*	A/G	Bates et al. 2011	intron
**rs7614913**	3	79567629	*ROBO1*	C/T	Bates et al. 2011	intron
**rs6548628**	3	79569558	*ROBO1*	A/C	Bates et al. 2011	intron
**rs7623728**	3	79597035	*ROBO1*	C/T	Bates et al. 2011	intron
**rs6548651**	3	79784416	*ROBO1*	A/G	Bates et al. 2011	intron
**rs1995402**	3	79790407	*ROBO1*	A/C	Bates et al. 2011	intron
**rs1365152**	3	139731266	*CLSTN2*	A/G	Roeske et al. 2011	intron
**rs2114167**	3	139779359	*CLSTN2*	G/T	Roeske et al. 2011	intron
**rs4234898**	4	156998541	*FTH1P21*	C/T	Roeske et al. 2011	-
**rs11100040**	4	157006954	*FTH1P21*	C/T	Roeske et al. 2011	-
**rs9356928**	6	24131335	*NRSN1*	A/G	Couto et al. 2010	intron
**rs1419228**	6	24178306	*DCDC2*	A/G	Lind et al. 2010; Paracchini et al. 2011	intron
**rs793862**	6	24207200	*DCDC2*	A/G	Meng et al. 2005; Schumacher et al. 2006; Ludwig et al. 2008; Wilcke et al. 2009; Scerri et al. 2011; Becker et al. 2013	intron
**rs9467076**	6	24209255	*DCDC2*	C/T	Lind et al. 2010	intron
**rs793842**	6	24224488	*DCDC2*	A/G	Darki et al. 2014	intron
**rs807701**	6	24273791	*DCDC2*	C/T	Schumacher et al. 2006; Ludwig et al. 2008; Wilcke et al. 2009; Becker et al. 2013	intron
**rs807724**	6	24278869	*DCDC2*	A/G	Meng et al. 2005	intron
**rs1091047**	6	24295256	*DCDC2*	C/G	Lind et al. 2010	intron
**rs7765678**	6	24330544	*DCDC2*	C/T	Lind et al. 2010	intron
**rs6922023**	6	24348117	*DCDC2*	A/G	Lind et al. 2010	intron
**rs1087266**	6	24355150	*DCDC2*	C/T	Meng et al. 2005	intron
**rs2179515**	6	24628203	*KIAA0319*	A/G	Cope et al. 2005; Becker et al. 2013	intron

SNP	Chr	Position	Gene	Type	Epidemiological evidence	SNP properties
**rs761100**	6	24632642	*KIAA0319*	G/T	Harold et al. 2006; Ludwig et al. 2008; Newbury et al. 2011; Becker et al. 2013	intron
**rs6935076**	6	24644322	*KIAA0319*	C/T	Cope et al. 2005; Luciano et al. 2007; Paracchini et al. 2008; Couto et al. 2010; Newbury et al. 2011; Scerri et al. 2011; Becker et al. 2013	intron
**rs3212236**	6	24648455	*TDP2*	A/G	Harold et al. 2006; Newbury et al. 2011	near gene
**rs9461045**	6	24649061	*TDP2*	C/T	Dennis et al. 2009; Newbury et al. 2011	near gene
**rs3181238**	6	24654443	*TDP2*	A/G	Couto et al. 2010	intron
**rs1842129**	6	124838090	*NKAIN2*	A/G	Luciano et al. 2011	intron
**rs923875**	7	113735036	*FOXP2*	A/C	Peter et al. 2011	intron
**rs12533005**	7	114056055	*FOXP2*	C/G	Peter et al. 2011; Wilcke et al. 2012	intron
**rs7782412**	7	114290415	*FOXP2*	C/T	Peter et al. 2011	intron
**rs936146**	7	114294405	*FOXP2*	C/G	Peter et al. 2011	intron
**rs706578**	7	137298910	*DGKI*	C/T	Matsson et al. 2011	intron
**rs270891**	7	137332959	*DGKI*	A/C	Matsson et al. 2011	intron
**rs270904**	7	137356651	*DGKI*	C/T	Matsson et al. 2011	intron
**rs1991084**	7	137398354	*DGKI*	A/G	Matsson et al. 2011	intron
**rs889869**	7	137506041	*DGKI*	A/G	Matsson et al. 2011	intron
**rs2351858**	7	137554435	*CREB3L2*	A/G	Matsson et al. 2011	-
**rs273933**	7	137577028	*CREB3L2*	A/G	Matsson et al. 2011	intron
**rs7794745**	7	146489606	*CNTNAP2*	A/T	Newbury et al. 2011	intron
**rs10246256**	7	147554807	*CNTNAP2*	C/T	Vernes et al. 2008; Newbury et al. 2011	intron
**rs2710102**	7	147574390	*CNTNAP2*	C/T	Vernes et al. 2008; Newbury et al. 2011; Peter et al. 2011; Whitehouse et al. 2011	intron
**rs759178**	7	147575112	*CNTNAP2*	G/T	Vernes et al. 2008; Whitehouse et al. 2011	intron
**rs17236239**	7	147582305	*CNTNAP2*	A/G	Vernes et al. 2008	intron
**rs2710117**	7	147601772	*CNTNAP2*	A/T	Vernes et al. 2008	intron
**rs9521789**	13	111119620	*COL4A2*	C/T	Eicher et al. 2013	intron
**rs8034835**	15	51512664	*CYP19A1*	A/G	Anthoni et al. 2012	intron
**rs730154**	15	51591204	*CYP19A1*	A/G	Anthoni et al. 2012	intron
**rs7174102**	15	55719687	*DYX1C1*	A/T	Paracchini et al. 2011	intron
**rs692691**	15	55760575	*DYX1C1*	C/T	Wigg et al. 2004	intron
**rs8037376**	15	55768321	*DYX1C1*	C/T	Paracchini et al. 2011	intron
**rs685935**	15	55770911	*DYX1C1*	C/T	Bates et al. 2010; Paracchini et al. 2011	intron
**rs11629841**	15	55777638	*DYX1C1*	G/T	Wigg et al. 2004	intron
**rs8043049**	15	55777788	*DYX1C1*	C/T	Paracchini et al. 2011	intron
**rs3743204**	15	55790310	*DYX1C1*	G/T	Wigg et al. 2004; Dahdouh et al. 2009; Bates et al. 2010; Becker et al. 2013	intron
**rs8040756**	15	55798599	*DYX1C1*	A/G	Paracchini et al. 2011	intron
**rs6564903**	16	81653657	*CMIP*	C/T	Newbury et al. 2009; Newbury et al. 2011; Scerri et al. 2011	intron
**rs3935802**	16	81661567	*CMIP*	C/G	Newbury et al. 2009; Newbury et al. 2011	intron
**rs4265801**	16	81665052	*CMIP*	G/T	Newbury et al. 2009	intron
**rs16955705**	16	81673350	*CMIP*	A/C	Scerri et al. 2011	intron

SNP	Chr	Position	Gene	Type	Epidemiological evidence	SNP properties
**rs7201632**	16	81677448	*CMIP*	C/T	Newbury et al. 2009; Newbury et al. 2011	intron
**rs8053211**	16	84453753	*ATP2C2*	A/G	Newbury et al. 2009; Newbury et al. 2011; Scerri et al. 2011	intron
**rs11860694**	16	84457447	*ATP2C2*	C/G	Newbury et al. 2009; Newbury et al. 2011	intron
**rs16973771**	16	84460578	*ATP2C2*	C/T	Newbury et al. 2009; Newbury et al. 2011	intron
**rs2875891**	16	84463909	*ATP2C2*	C/T	Newbury et al. 2009; Newbury et al. 2011	intron
**rs8045507**	16	84464577	*ATP2C2*	A/G	Newbury et al. 2009; Newbury et al. 2011	intron
**rs11874896**	18	5622071	*EPB41L3*	A/T	Scerri et al. 2010	intron
**rs1299348**	18	13822256	*AP001525.1*	A/G	Scerri et al. 2010	near gene
**rs10502812**	18	41060918	*SYT4*	A/G	Scerri et al. 2010	-
**rs11661879**	18	46374076	*CTIF*	C/T	Scerri et al. 2010	intron
**rs11873029**	18	46617055	*DYM*	C/T	Scerri et al. 2010	intron
**rs8094327**	18	55963045	*NEDD4L*	A/G	Scerri et al. 2010; Mueller et al. 2014	intron
**rs12606138**	18	55993944	*NEDD4L*	A/G	Scerri et al. 2010; Mueller et al. 2014	intron
**rs4807927**	19	1423199	*DAZAP1*	A/G	Luciano et al. 2013	intron
**rs2192161**	21	15681994	*ABCC13*	C/T	Luciano et al. 2013	intron

Shown are the SNPs with the respective position (hg19), the respective gene (GRCh37), the SNP-type, the reference for the epidemiological evidence and properties of the SNP.

References

Anthoni H, Sucheston LE, Lewis BA, Tapia-Páez I, Fan X, Zucchelli M, Taipale M, Stein CM, Hokkanen M-E, Castrén E, *et al.* (2012) The aromatase gene CYP19A1: several genetic and functional lines of evidence supporting a role in reading, speech and language. Behav Genet 42:509-527. doi: 10.1007/s10519-012-9532-3.

Anthoni H, Zucchelli M, Matsson H, Müller-Myhsok B, Fransson I, Schumacher J, Massinen S, Onkamo P, Warnke A, Griesemann H, *et al.* (2007) A locus on 2p12 containing the co-regulated MRPL19 and C2ORF3 genes is associated to dyslexia. Hum Mol Genet 16:667-677. doi: 10.1093/hmg/ddm009.

Bates TC, Lind PA, Luciano M, Montgomery GW, Martin NG and Wright MJ (2010) Dyslexia and DYX1C1: deficits in reading and spelling associated with a missense mutation. Mol Psychiatry 15:1190-1196. doi: 10.1038/mp.2009.120.

Bates TC, Luciano M, Medland SE, Montgomery GW, Wright MJ and Martin NG (2011) Genetic variance in a component of the language acquisition device: ROBO1 polymorphisms associated with phonological buffer deficits. Behav Genet 41:50-57. doi: 10.1007/s10519-010-9402-9.

Becker J, Czamara D, Scerri TS, Ramus F, Csépe V, Talcott JB, Stein J, Morris A, Ludwig KU, Hoffmann P, *et al.* (2013) Genetic analysis of dyslexia candidate genes in the European cross-linguistic NeuroDys cohort. Eur J Hum Genet 1-6. doi: 10.1038/ejhg.2013.199.

Cope N, Harold D, Hill G, Moskvina V, Stevenson J, Holmans P, Owen MJ, O'Donovan MC and Williams J (2005) Strong evidence that KIAA0319 on chromosome 6p is a susceptibility gene for developmental dyslexia. Am J Hum Genet 76:581-591. doi: 10.1086/429131.

Couto JM, Livne-Bar I, Huang K, Xu Z, Cate-Carter T, Feng Y, Wigg K, Humphries T, Tannock R, Kerr EN, *et al.* (2010) Association of reading disabilities with regions marked by acetylated H3 histones in KIAA0319. Am J Med Genet B Neuropsychiatr Genet 153B:447-462. doi: 10.1002/ajmg.b.30999.

Dahdouh F, Anthoni H, Tapia-Páez I, Peyrard-Janvid M, Schulte-Körne G, Warnke A, Remschmidt H, Ziegler A, Kere J, Müller-Myhsok B, *et al.* (2009) Further evidence for DYX1C1 as a susceptibility factor for dyslexia. Psychiatr Genet 19:59-63. doi: 10.1097/YPG.0b013e32832080e1.

Darki F, Peyrard-Janvid M, Matsson H, Kere J and Klingberg T (2014) DCDC2 Polymorphism Is Associated with Left Temporoparietal Gray and White Matter Structures during Development. J Neurosci 34:14455-14462. doi: 10.1523/JNEUROSCI.1216-14.2014.

Dennis MY, Paracchini S, Scerri TS, Prokunina-Olsson L, Knight JC, Wade-Martins R, Coggill P, Beck S, Green ED and Monaco AP (2009) A common variant associated with dyslexia reduces expression of the KIAA0319 gene. PLoS Genet 5:e1000436. doi: 10.1371/journal.pgen.1000436.

Eicher JD, Powers NR, Miller LL, Akshoomoff N, Amaral DG, Bloss CS, Libiger O, Schork NJ, Darst BF, Casey BJ, *et al.* (2013) Genome-wide association study of shared components of reading disability and language impairment. Genes Brain Behav 12:792-801. doi: 10.1111/gbb.12085.

Harlaar N, Butcher LM, Meaburn E, Sham P, Craig IW and Plomin R (2005) A behavioural genomic analysis of DNA markers associated with general cognitive ability in 7-year-olds. J Child Psychol Psychiatry 46:1097-107. doi: 10.1111/j.1469-7610.2005.01515.x.

Harold D, Paracchini S, Scerri T, Dennis M, Cope N, Hill G, Moskvina V, Walter J, Richardson AJ, Owen MJ, *et al.* (2006) Further evidence that the KIAA0319 gene confers susceptibility to developmental dyslexia. Mol Psychiatry 11:1085-1091, 1061. doi: 10.1038/sj.mp.4001904.

Lind PA, Luciano M, Wright MJ, Montgomery GW, Martin NG and Bates TC (2010) Dyslexia and DCDC2: normal variation in reading and spelling is associated with DCDC2 polymorphisms in an Australian population sample. Eur J Hum Genet 18:668-673. doi: 10.1038/ejhg.2009.237.

Luciano M, Evans DM, Hansell NK, Medland SE, Montgomery GW, Martin NG, Wright MJ and Bates TC (2013) A genome-wide association study for reading and language abilities in two population cohorts. Genes Brain Behav 12:645-652. doi: 10.1111/gbb.12053.

Luciano M, Lind PA, Duffy DL, Castles A, Wright MJ, Montgomery GW, Martin NG and Bates TC (2007) A haplotype spanning KIAA0319 and TTRAP is associated with normal variation in reading and spelling ability. Biol Psychiatry 62:811-817. doi: 10.1016/j.biopsych.2007.03.007.

Luciano M, Montgomery GW, Martin NG, Wright MJ and Bates TC (2011) SNP sets and reading ability: testing confirmation of a 10-SNP set in a population sample. Twin Res Hum Genet 14:228-232. doi: 10.1375/twin.14.3.228.

Ludwig KU, Roeske D, Schumacher J, Schulte-Körne G, König IR, Warnke A, Plume E, Ziegler A, Remschmidt H, Müller-Myhsok B, *et al.* (2008) Investigation of interaction between DCDC2 and KIAA0319 in a large German dyslexia sample. J Neural Transm 115:1587-1589. doi: 10.1007/s00702-008-0124-6.

Matsson H, Tammimies K, Zucchelli M, Anthoni H, Onkamo P, Nopola-Hemmi J, Lyytinen H, Leppanen PHT, Neuhoff N, Warnke A, *et al.* (2011) SNP variations in the 7q33 region containing DGKI are associated with dyslexia in the Finnish and German populations. Behav Genet 41:134-140. doi: 10.1007/s10519-010-9431-4.

Meng H, Smith SD, Hager K, Held M, Liu J, Olson RK, Pennington BF, DeFries JC, Gelernter J, O'Reilly-Pol T, *et al.* (2005) DCDC2 is associated with reading disability and modulates neuronal development in the brain. Proc Natl Acad Sci U S A 102:17053-17058. doi: 10.1073/pnas.0508591102.

Newbury DF, Paracchini S, Scerri TS, Winchester L, Addis L, Richardson AJ, Walter J, Stein JF, Talcott JB and Monaco AP (2011) Investigation of dyslexia and SLI risk variants in reading- and language-impaired subjects. Behav Genet 41:90-104. doi: 10.1007/s10519-010-9424-3.

Newbury DF, Winchester L, Addis L, Paracchini S, Buckingham L-L, Clark A, Cohen W, Cowie H, Dworzynski K, Everitt A, *et al.* (2009) CMIP and ATP2C2 modulate phonological short-term memory in language impairment. Am J Hum Genet 85:264-272. doi: 10.1016/j.ajhg.2009.07.004.

Paracchini S, Ang QW, Stanley FJ, Monaco AP, Pennell CE and Whitehouse AJO (2011) Analysis of dyslexia candidate genes in the Raine cohort representing the general Australian population. Genes Brain Behav 10:158-165. doi: 10.1111/j.1601-183X.2010.00651.x.

Paracchini S, Steer CD, Buckingham L-L, Morris AP, Ring S, Scerri T, Stein J, Pembrey ME, Ragoussis J, Golding J, *et al.* (2008) Association of the KIAA0319 dyslexia susceptibility gene with reading skills in the general population. Am J Psychiatry 165:1576-1584. doi: 10.1176/appi.ajp.2008.07121872.

Peter B, Raskind WH, Matsushita M, Lisowski M, Vu T, Berninger VW, Wijsman EM and Brkanac Z (2011) Replication of CNTNAP2 association with nonword repetition and support for FOXP2 association with timed reading and motor activities in a dyslexia family sample. J Neurodev Disord 3:39-49. doi: 10.1007/s11689-010-9065-0.

Roeske D, Ludwig KU, Neuhoff N, Becker J, Bartling J, Bruder J, Brockschmidt FF, Warnke A, Remschmidt H, Hoffmann P, *et al.* (2011) First genome-wide association scan on neurophysiological endophenotypes points to trans-regulation effects on SLC2A3 in dyslexic children. Mol Psychiatry 16:97-107. doi: 10.1038/mp.2009.102.

Scerri TS, Morris AP, Buckingham LL, Newbury DF, Miller LL, Monaco AP, Bishop DVM and Paracchini S (2011) DCDC2, KIAA0319 and CMIP are associated with reading-related traits. Biol Psychiatry 70:237-245. doi: 10.1016/j.biopsych.2011.02.005.

Scerri TS, Paracchini S, Morris A, MacPhie IL, Talcott J, Stein J, Smith SD, Pennington BF, Olson RK, DeFries JC, *et al.* (2010) Identification of candidate genes for dyslexia susceptibility on chromosome 18. PLoS One 5:e13712. doi: 10.1371/journal.pone.0013712.

Schumacher J, Anthoni H, Dahdouh F, König IR, Hillmer AM, Kluck N, Manthey M, Plume E, Warnke A, Remschmidt H, *et al.* (2006) Strong genetic evidence of DCDC2 as a susceptibility gene for dyslexia. Am J Hum Genet 78:52-62. doi: 10.1086/498992.

Tran C, Wigg KG, Zhang K, Cate-Carter TD, Kerr E, Field LL, Kaplan BJ, Lovett MW and Barr CL (2014) Association of the ROBO1 gene with reading disabilities in a family-based analysis. Genes Brain Behav 13:430-438. doi: 10.1111/gbb.12126.

Vernes SC, Newbury DF, Abrahams BS, Winchester L, Nicod J, Groszer M, Alarcón M, Oliver PL, Davies KE, Geschwind DH, *et al.* (2008) A functional genetic link between distinct developmental language disorders. N Engl J Med 359:2337-2345. doi: 10.1056/NEJMoa0802828.

Whitehouse AJO, Bishop DVM, Ang QW, Pennell CE and Fisher SE (2011) CNTNAP2 variants affect early language development in the general population. Genes Brain Behav 10:451-456. doi: 10.1111/j.1601-183X.2011.00684.x.

Wigg KG, Couto JM, Feng Y, Anderson B, Cate-Carter TD, Macciardi F, Tannock R, Lovett MW, Humphries TW and Barr CL (2004) Support for EKN1 as the susceptibility locus for dyslexia on 15q21. Mol Psychiatry 9:1111-1121. doi: 10.1038/sj.mp.4001543.

Wilcke A, Ligges C, Burkhardt J, Alexander M, Wolf C, Quente E, Ahnert P, Hoffmann P, Becker A, Müller-Myhsok B, *et al.* (2012) Imaging genetics of FOXP2 in dyslexia. Eur J Hum Genet 20:224-229. doi: 10.1038/ejhg.2011.160.

Wilcke A, Weissfuss J, Kirsten H, Wolfram G, Boltze J and Ahnert P (2009) The role of gene DCDC2 in German dyslexics. Ann Dyslexia 59:1-11. doi: 10.1007/s11881-008-0020-7.
