# Supplementary material for: Dyslexia risk variant rs600753 is linked with dyslexia-specific differential allelic expression of DYX1C1
Source: Genet Mol Biol. 2018 Feb 19;41(1):41–9. doi: 10.1590/1678-4685-GMB-2017-0165 (PMC5901500; doi:10.1590/1678-4685-GMB-2017-0165)
Supplement: Supplementary file 2 [file 1415-4757-GMB-41-01-2017-0165-s003.pdf]

Supplementary material to "Dyslexia risk variant rs600753 is linked with dyslexia-specific differential allelic expression of *DYX1C1*"

Table S3 - Primer sequences.

SNP	Direction	cDNA Sequence	gDNA Sequence	SBE Primer
rs3743205	F	TGGAGGAAAGTTGACCTTCAGA		
	R	GGTAACCCCAGCTTCCCTAG		
rs10046	F	TGGCAAGGATGGATGATTTGT	TGGTGTGAACAGGAGCAGAT	bioAACACTAGAGAAGGCTG[L]TCAGTACC
	R	CTTGAAAATTTTGCAAAGAATGTTCC	CCCCAAGAAACTCAGACAGG	
rs600753	F	TTTTCTGAATTTCTCCCTTTTG	ACTTCACCAACAGGACTCACT	bioGTCAAAAAGAAAAGCA[L]ATTAAAGAAG
	R	ATGAACGGATAAAAGCCACT	ACAATGGCAAGAGTTTAGAGGT	
rs6564903	F	GGACTGGATGATGGCAATGG		
	R	CTATTGGCAGCGGGAGGG		
rs9467075	F	ACATCAGCCTCATCTTGTCCA	GGTTGATCAGCCACATAGTACC	bioAAGATACTCAGGT[L]GAGGTTCCAGT
	R	AGAGAGGTCTGAAACACGGG	AGAGAGGTCTGAAACACGGG	
rs555879	F	TGCAAAGATCAAAACTAGGCAG		
	R	ACGAACAATCCAGGCACAAC		
rs934634	F	AGAGAGCTTGCTGGATGTGA	AGAGAGCTTGCTGGATGTGA	bioCAATG[L]AGGCATGGGGGTGT
	R	GTGCTTAACCGTCACTCTTGA	TGCCCCATTGTTAAGTGCTT	

cDNA specific primers for seven SNPs were constructed to test the SNP expression in blood cells. Based on the results, gDNA-specific primers and SBE primers were constructed for four SNPs in order to analyze DAE. Primers highlighted in gray were used for Sanger sequencing.
