# Supplementary material for: Dyslexia risk variant rs600753 is linked with dyslexia-specific differential allelic expression of DYX1C1
Source: Genet Mol Biol. 2018 Feb 19;41(1):41–9. doi: 10.1590/1678-4685-GMB-2017-0165 (PMC5901500; doi:10.1590/1678-4685-GMB-2017-0165)
Supplement: Supplementary file 3 [file 1415-4757-GMB-41-01-2017-0165-s004.pdf]

Supplementary material to "Dyslexia risk variant rs600753 is linked with dyslexia-specific differential allelic expression of *DYX1C1*"

Table S4 - Number of sequences surviving quality control.

**rs600753**	**F**	**R**	**rs10046**	**F**	**R**
Dyslexia	6	6	Dyslexia	6	6
Controls	7	5	Controls	9	9
**rs93446**	**F**	**R**	**rs9467075**	**F**	**R**
Dyslexia	4	4	Dyslexia	5	4
Controls	5	5	Controls	7	5
