# Supplementary material for: Dyslexia risk variant rs600753 is linked with dyslexia-specific differential allelic expression of DYX1C1
Source: Genet Mol Biol. 2018 Feb 19;41(1):41–9. doi: 10.1590/1678-4685-GMB-2017-0165 (PMC5901500; doi:10.1590/1678-4685-GMB-2017-0165)
Supplement: Table S1 [file 1415-4757-GMB-41-01-2017-0165-s001.pdf]

Supplementary material to "Dyslexia risk variant rs600753 is linked with dyslexia-specific differential allelic expression of *DYX1C1*"

Table S1 - Overview of considered SNPs.

SNP	Chr	Position	Gene	Type	Epidemiological evidence	SNP properties	Expressed in B-cells	Heterozygous lines (dyslexia family & controls)	DAE analyzed
**rs3178**	6	24147572	*NRSN1*	C/T	Couto et al. 2010	3'UTR	not tested	-	-
**rs9467075**	6	24205236	*DCDC2*	A/G	Lind et al. 2010	exon	yes	5 & 8	analyzed
**rs4504469**	6	24588884	*KIAA0319*	C/G/T	Francks et al. 2004; Cope et al. 2005; Luciano et al. 2007; Paracchini et al. 2008; Newbury et al. 2011; Becker et al. 2013	exon	not tested	-	-
**rs2038137**	6	24645943	*KIAA0319*	A/C	Francks et al. 2004; Luciano et al. 2007; Paracchini et al. 2008; Paracchini et al. 2011	5'UTR	not tested	-	-
**rs2143340**	6	24659071	*TDP2*	C/T	Francks et al. 2004; Luciano et al. 2007; Paracchini et al. 2008; Newbury et al. 2011; Paracchini et al. 2011	non-coding exon	no	4 & 7	-
**rs3734972**	7	128470838	*FLNC*	C/T	Gialluisi et al. 2014	exon	not tested	-	-
**rs934634**	15	51500538	*CYP19A1*	C/T	Anthoni et al. 2012	3'UTR	yes	4 & 9	analyzed
**rs10046**	15	51502986	*CYP19A1*	C/T	Anthoni et al. 2012	3'UTR	yes	6 & 11	analyzed
**rs600753**	15	55759193	*DYX1C1*	C/T	Dahdouh et al. 2009	exon	yes	7 & 10	analyzed
**rs17819126**	15	55789910	*DYX1C1*	C/T	Bates et al. 2010; Paracchini et al. 2011	exon	not tested	0 & 1	-
**rs3743205**	15	55790530	*DYX1C1*	A/C/T	Taipale et al. 2003; Wigg et al. 2004; Dahdouh et al. 2009; Lim et al. 2011; Newbury et al. 2011; Becker et al. 2013	5'UTR	no	6 & 1	-
**rs555879**	18	47352533	*MYO5B*	C/T	Scerri et al. 2010; Mueller et al. 2014	3'UTR	no	7 & 14	-

Shown are the SNPs with the respective position (hg19), the respective gene (GRCh37), the SNP-type, the reference for the epidemiological evidence, properties of the SNP, the number of available cell lines and if they were analyzed.

References

Anthoni H, Sucheston LE, Lewis BA, Tapia-Páez I, Fan X, Zucchelli M, Taipale M, Stein CM, Hokkanen M-E, Castrén E, *et al.* (2012) The aromatase gene CYP19A1: several genetic and functional lines of evidence supporting a role in reading, speech and language. Behav Genet 42:509-527. doi: 10.1007/s10519-012-9532-3.

Bates TC, Lind PA, Luciano M, Montgomery GW, Martin NG and Wright MJ (2010) Dyslexia and DYX1C1: deficits in reading and spelling associated with a missense mutation. Mol Psychiatry 15:1190-1196. doi: 10.1038/mp.2009.120.

Becker J, Czamara D, Scerri TS, Ramus F, Csépe V, Talcott JB, Stein J, Morris A, Ludwig KU, Hoffmann P, *et al.* (2013) Genetic analysis of dyslexia candidate genes in the European cross-linguistic NeuroDys cohort. Eur J Hum Genet 1-6. doi: 10.1038/ejhg.2013.199.

Cope N, Harold D, Hill G, Moskvina V, Stevenson J, Holmans P, Owen MJ, O'Donovan MC and Williams J (2005) Strong evidence that KIAA0319 on chromosome 6p is a susceptibility gene for developmental dyslexia. Am J Hum Genet 76:581-591. doi: 10.1086/429131.

Couto JM, Livne-Bar I, Huang K, Xu Z, Cate-Carter T, Feng Y, Wigg K, Humphries T, Tannock R, Kerr EN, *et al.* (2010) Association of reading disabilities with regions marked by acetylated H3 histones in KIAA0319. Am J Med Genet B Neuropsychiatr Genet 153B:447-462. doi: 10.1002/ajmg.b.30999.

Dahdouh F, Anthoni H, Tapia-Páez I, Peyrard-Janvid M, Schulte-Körne G, Warnke A, Remschmidt H, Ziegler A, Kere J, Müller-Myhsok B, *et al.* (2009) Further evidence for DYX1C1 as a susceptibility factor for dyslexia. Psychiatr Genet 19:59-63. doi: 10.1097/YPG.0b013e32832080e1.

Francks C, Paracchini S, Smith SD, Richardson AJ, Scerri TS, Cardon LR, Marlow AJ, MacPhie IL, Walter J, Pennington BF, *et al.* (2004) A 77-kilobase region of chromosome 6p22.2 is associated with dyslexia in families from the United Kingdom and from the United States. Am J Hum Genet 75:1046-1058. doi: 10.1086/426404.

Gialluisi A, Newbury DF, Wilcutt EG, Olson RK, DeFries JC, Brandler WM, Pennington BF, Smith SD, Scerri TS, Simpson NH, *et al.* (2014) Genome-wide screening for DNA variants associated with reading and language traits. Genes Brain Behav 13:686-701. doi: 10.1111/gbb.12158.

Lim CKP, Ho CSH, Chou CHN and Waye MMY (2011) Association of the rs3743205 variant of DYX1C1 with dyslexia in Chinese children. Behav Brain Funct 7:16. doi: 10.1186/1744-9081-7-16.

Lind PA, Luciano M, Wright MJ, Montgomery GW, Martin NG and Bates TC (2010) Dyslexia and DCDC2: normal variation in reading and spelling is associated with DCDC2 polymorphisms in an Australian population sample. Eur J Hum Genet 18:668-673. doi: 10.1038/ejhg.2009.237.

Luciano M, Lind PA, Duffy DL, Castles A, Wright MJ, Montgomery GW, Martin NG and Bates TC (2007) A haplotype spanning KIAA0319 and TTRAP is associated with normal variation in reading and spelling ability. Biol Psychiatry 62:811-817. doi: 10.1016/j.biopsych.2007.03.007.

Mueller B, Ahnert P, Burkhardt J, Brauer J, Czepezauer I, Quente E, Boltze J, Wilcke A and Kirsten H (2014) Genetic risk variants for dyslexia on chromosome 18 in a German cohort. Genes Brain Behav 13:350-356. doi: 10.1111/gbb.12118.

Newbury DF, Paracchini S, Scerri TS, Winchester L, Addis L, Richardson AJ, Walter J, Stein JF, Talcott JB and Monaco AP (2011) Investigation of dyslexia and SLI risk variants in reading- and language-impaired subjects. Behav Genet 41:90-104. doi: 10.1007/s10519-010-9424-3.

Paracchini S, Ang QW, Stanley FJ, Monaco AP, Pennell CE and Whitehouse AJO (2011) Analysis of dyslexia candidate genes in the Raine cohort representing the general Australian population. Genes Brain Behav 10:158-165. doi: 10.1111/j.1601-183X.2010.00651.x.

Paracchini S, Steer CD, Buckingham L-L, Morris AP, Ring S, Scerri T, Stein J, Pembrey ME, Ragoussis J, Golding J, *et al.* (2008) Association of the KIAA0319 dyslexia susceptibility gene with reading skills in the general population. Am J Psychiatry 165:1576-1584. doi: 10.1176/appi.ajp.2008.07121872.

Scerri TS, Paracchini S, Morris A, MacPhie IL, Talcott J, Stein J, Smith SD, Pennington BF, Olson RK, DeFries JC, *et al.* (2010) Identification of candidate genes for dyslexia susceptibility on chromosome 18. PLoS One 5:e13712. doi: 10.1371/journal.pone.0013712.

Taipale M, Kaminen N, Nopola-Hemmi J, Haltia T, Myllyluoma B, Lyytinen H, Muller K, Kaaranen M, Lindsberg PJ, Hannula-Jouppi K, *et al.* (2003) A candidate gene for developmental dyslexia encodes a nuclear tetratricopeptide repeat domain protein dynamically regulated in brain. Proc Natl Acad Sci U S A 100:11553-11558. doi: 10.1073/pnas.1833911100.

Wigg KG, Couto JM, Feng Y, Anderson B, Cate-Carter TD, Macciardi F, Tannock R, Lovett MW, Humphries TW and Barr CL (2004) Support for EKN1 as the susceptibility locus for dyslexia on 15q21. Mol Psychiatry 9:1111-1121. doi: 10.1038/sj.mp.4001543.
