# Supplementary material for: Dyslexia risk variant rs600753 is linked with dyslexia-specific differential allelic expression of DYX1C1
Source: Genet Mol Biol. 2018 Feb 19;41(1):41–9. doi: 10.1590/1678-4685-GMB-2017-0165 (PMC5901500; doi:10.1590/1678-4685-GMB-2017-0165)
Supplement: Figure S1 [file 1415-4757-GMB-41-01-2017-0165-s005.pdf]

Supplementary material to "Dyslexia risk variant rs600753 is linked with dyslexia-specific differential allelic expression of *DYX1C1*"

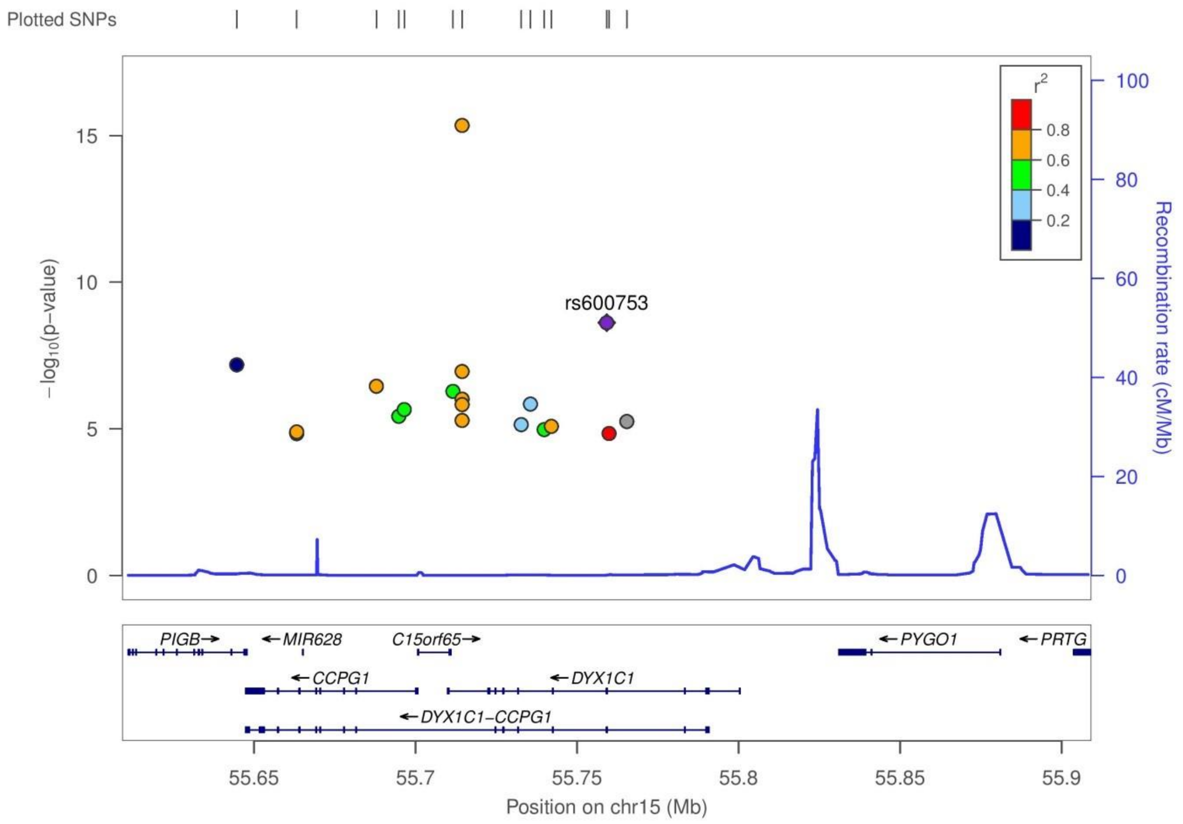

Figure S1 - Local association plot of rs600753. Previously reported *cis* eQTLs affecting the expression levels of *DYX1C1* are shown together with their respective p-values. All eQTLs were colored according to their linkage to rs600753. The SNP with the lowest p-value is rs12324434, however, in contrast to rs600753 this variant was not found to be associated with dyslexia (Bates et al. 2010; Paracchini et al. 2011).

References

Bates TC, Lind PA, Luciano M, Montgomery GW, Martin NG and Wright MJ (2010) Dyslexia and DYX1C1: deficits in reading and spelling associated with a missense mutation. Mol Psychiatry 15:1190-1196. doi: 10.1038/mp.2009.120.

Paracchini S, Ang QW, Stanley FJ, Monaco AP, Pennell CE and Whitehouse AJO (2011) Analysis of dyslexia candidate genes in the Raine cohort representing the general Australian population. Genes Brain Behav 10:158-165. doi: 10.1111/j.1601-183X.2010.00651.x.
