# Supplementary material for: Dyslexia risk variant rs600753 is linked with dyslexia-specific differential allelic expression of DYX1C1
Source: Genet Mol Biol. 2018 Feb 19;41(1):41–9. doi: 10.1590/1678-4685-GMB-2017-0165 (PMC5901500; doi:10.1590/1678-4685-GMB-2017-0165)
Supplement: Figure S2 [file 1415-4757-GMB-41-01-2017-0165-s006.pdf]

Supplementary material to "Dyslexia risk variant rs600753 is linked with dyslexia-specific differential allelic expression of *DYX1C1*"

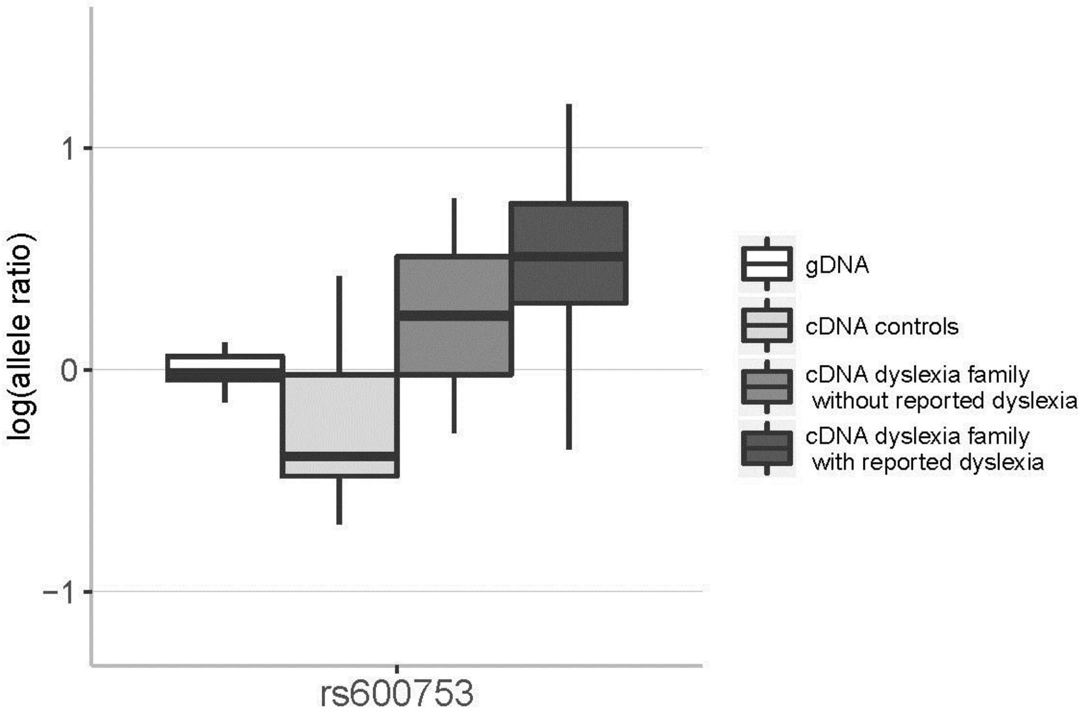

Figure S2 - The effect of rs600753 stratified for gDNA, cDNA from control cell lines (N=10), cDNA from dyslexia family members without reported dyslexia (N=2), and cDNA from dyslexia family members with reported dyslexia (N=5). The Kruskal-Wallis test detected trend-level significance (p=0.053).
